# Community activity intervention reduced sitting time and improved population physical activity levels

**DOI:** 10.1186/s12889-025-26105-6

**Published:** 2026-01-06

**Authors:** Robert C. MacKinnon, Zoe Marshall, Steve Rose, Matthew Jewiss, Laurie Butler, Julia Gawronska, Lee Smith, William Bird, Gregory Deacon

**Affiliations:** 1 Anglia Ruskin University, East Road, Cambridge, UK; 2 Intelligent Health Ltd, Reading Enterprise Centre, University of Reading, Reading, UK; 3 Institute of Sport, Manchester Metropolitan University, Manchester, UK; 4 Health Determinants Research Collaboration Greater Essex, Essex County Council, Chelmsford, UK; 5 Centre for Health, Performance and Wellbeing, Anglia Ruskin University, Cambridge, UK; 6 Department of Public Health, Faculty of Medicine, Biruni University, Istanbul, Turkey; 7 Faculty of Health and Life Sciences, University of Exeter, Exeter, UK

**Keywords:** Physical activity, Gamification, Behavioural change

## Abstract

**Background:**

The aim of the present study was to investigate the impact of the Beat the Street intervention; a gamification intervention to promote physical activity, reduce sitting time and improve wellbeing, among residents of Chelmsford and South Woodham Ferrers.

**Methods:**

Pre-post experimental design of the Beat the Street Intervention. Beat the Street gamifies neighbourhoods, incentivising the community to actively travel, collecting virtual points at sensors on selected lamp posts called ‘Beat Boxes’ advertised on maps and phone applications. Participants were asked to complete a baseline questionnaire after registering, and before the six-week “game phase” of the intervention. A follow-up questionnaire was then completed at the end of the intervention. Mean differences were calculated to provide interpretations of differences. Within-subjects differences for all measures were assessed by Wilcoxon Signed Rank separately for children and adults, followed by each demographic grouping. Significance for all analyses was set at *p* < 0.05.

**Results:**

Nine hundred fifteen (*n* = 313 children) participants reported sitting time and moderate-to-vigorous physical activity both pre- and post-intervention. For both children and adults overall, there was an increase in reported wellbeing and a reduction in reported sitting time daily and sitting time week, while life satisfaction increased for adults overall. Additionally, the percentage of participants classed as inactive decreased for both children and adults.

**Conclusion:**

The present findings suggest that the Beat the Street intervention reduced sitting time and increased physical activity among both children and adults in the short term. Further research is needed to assess the long-term impacts and explore objective measures of sitting time to better understand sustained engagement and outcomes.

## Introduction

Regular and sustained participation in physical activity is associated with almost every facet of physical, cognitive, affective and social health across the lifespan and, importantly, the prevention of all-cause early mortality [1, 2]. Physical activity is defined as any bodily movement produced by skeletal muscle that results in energy expenditure [3]. Sedentary behaviour can be defined as any waking behaviour with an energy expenditure of ≤ 1.5 Metabolic Equivalents (METs) while in a sitting or reclining posture [4], which includes watching TV, video gaming, passive transport [car, bus, train] and computer use. It has been established that sedentary behaviour, independent of physical activity levels, is negatively associated with physical, mental health and social outcomes [5]. For instance, sedentary behaviour is associated with all-cause mortality, cardiovascular diseases as well as mood disorders like depression and cognitive impairment. Given the overwhelming health benefits of regular and sustained participation in physical activity and low levels of sedentary time, governments have produced physical activity recommendations for their populations. The UK Chief Medical Officer’s guidelines state that adults should achieve at least 150 min of moderate intensity physical activity or at least 75 min of vigorous intensity physical activity (or a combination of the two) per week, to engage in muscle strengthening activities twice a week and to minimise sedentary time, whereas children and adolescents are encouraged to aim for a minimum average of 60 min of physical activity a day across the week through a range of activities (e.g., playing, running, swimming, skipping and dancing) and to minimise sedentary time [6].

Within the UK population levels of physical activity are low and sedentary behaviour high across all age groups. Just 63.1% of the adult population [7], and 44.6% of children and young people in school years 1–11 (aged 5–16) in England met the Chief Medical Officers’ guidelines in 2020–21 [8]. Moreover, adults and children sit for a significant proportion of their day; 74% of adults in their mid-40 s have been estimated to sit for more than eight hours per day, and 25% of this time was made up of prolonged bouts of sitting for an hour or more at a time [9] while children in the UK have also been reported to sit for excessive amounts of time [10].

Against the backdrop of vast physical, mental and social benefits to achieving adequate physical activity and low sedentary time, and the risks associated with inadequate physical activity and high sedentary time, community interventions are urgently needed to address both increasing physical activity and reducing sedentary behaviour across all age groups. In contrast to individual programmes and initiatives designed to promote physical activity, community interventions are more achievable at a regional rather than national level, where variation in physical and sedentary activity figures is known to be heavily predicated on local factors. For example, the county of Essex is ranked below the national average for the proportion of individuals not meeting the physical activity guidelines [11]. Moreover, within Essex there are stark disparities in levels of physical activity within the town of Chelmsford which is ranked among the inner county regions with the lowest levels of physical activity [11].

One potential approach to increasing physical activity levels and reducing sedentary time is via active travel (e.g., walking, cycling, scooting etc.) or the promotion of walking and cycling [12]. Research has shown that adults who used > 60 min of active travel to their destination accumulated significantly more total physical activity than those who reported no active travel. Similarly, in children, research has demonstrated that children who walk or cycle to school are more likely to be more physically active in extra-curricular hours than those who take a non-active form of transport [13]. More specifically, research has shown children who walk or cycle to school are likely to engage in around six minutes more moderate to vigorous physical activity (MVPA) a day which equates to approximately 40% of daily average minutes of MVPA a day [14].

On a mechanistic level, interventions that aim to increase active travel in all age groups by nature displace sedentary activities with physical activity during a commute per se. Moreover, the commute offers opportunities for children and young people to engage in spontaneous and autonomous play. In addition, children and young people can develop a sense of relatedness by commuting and subsequently playing with their peer group [15, 16].

Physically active approaches to journeying to school or work can also be incentivised to encourage participation. The concept of gamification is emerging in the academic literature as a promising tool to increase population levels of physical activity and potentially reduce sedentary time [17, 18]. Here, it has been shown that gamified interventions are associated with a significant proportion of additional physical activity than inactive control groups and active control groups which did not contain a gamified physical activity intervention. Moreover, a key barrier to active travel per se is lack of motivation [19, 20] and gamification may be able to address this barrier by making active travel more engaging, rewarding, and social.

**Fig. 1:**
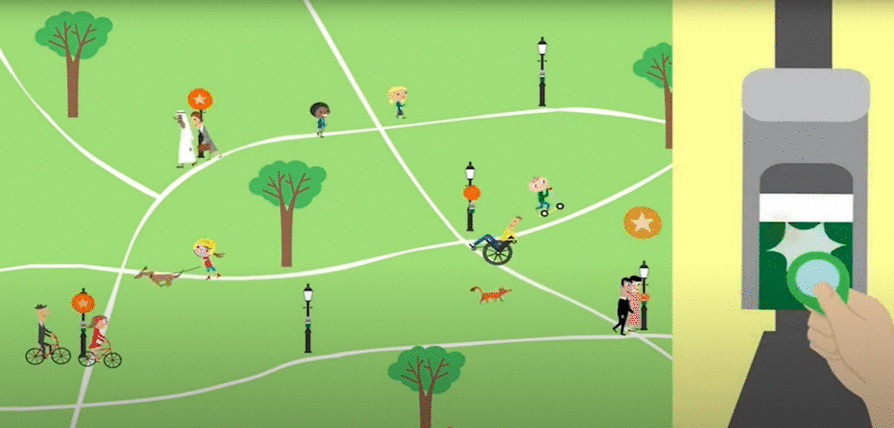
Screenshot from introduction video for the Beat the Street intervention, showing interaction with lamppost-based “beat boxes”

Given the benefits of active travel and gamification and in order to encourage greater walking and cycling amongst residents either in their commute to work/school and/or in exploration of the local area, a Beat the Street initiative was commissioned across Chelmsford and South Woodham Ferrers [21]. The initiative (Fig. 1) works across neighbourhoods, incentivising communities to travel actively by collecting points for themselves and their community teams at RFID sensors called ‘Beat Boxes’. These are placed strategically creating a network of publicly accessible locations, such as on lamp posts, in parks, outside schools, and along active-mobility infrastructure, all of which are shown on physical maps and in the mobile app. Players can play individually but can also come together to form community groups to collect points as a team. School children can do the same, with points gained by each child contributing to a school’s leaderboard over the six weeks of the game. Each week is themed, maximising engagement strategies and rewarding positive behaviour change that has been shown to increase as the intervention progresses as well as continuing after its six-week completion.

Consequently, the aim of the present study was to understand and address low levels of physical activity and high levels of sedentary behaviour (i.e. sitting time) by evaluating a community targeted intervention based within Essex, specifically focused on Chelmsford and South Woodham Ferrers. Specifically, we investigate the impact of the Beat the Street intervention on residents of Chelmsford and South Woodham Ferrers across all age groups utilising a pre-post intervention experimental design.

## Methods

### Study setting and population

The theory of change, behavioural basis and in-depth description of the Beat the Street intervention has been described previously [22]. The Beat the Street community intervention was collaboratively conducted in Chelmsford and South Woodham Ferrers by the digital health company, Intelligent Health UK, and co-funded by Anglia Ruskin University, Essex County Council, Active Essex, Chelmsford City Council and Sport England. The intervention was six weeks in duration from February until April 2024. Participants scanned physical cards on radio frequency identification readers, Beat Boxes, or phones using a custom phone Application with the aim of gaining points. Ten points were gained if two different Beat Boxes were scanned within an hour of one another. Beat Boxes were mapped across Chelmsford and South Woodham Ferrers, located along active travel routes and in green spaces, with a minimum of 500 m between each Beat Box. The highest scoring adult and child participants, community group and school were rewarded with prizes themed around active lifestyle.

Participation in *Beat the Street* was free of charge. All primary school children received a participation pack through their school, which included a letter, game cards, and an engaging printed map showing the locations of Beat Boxes. Children took this pack home to encourage parental involvement, and parents registered themselves and their children to take part online. The programme was promoted locally through digital channels and printed materials (e.g., leaflets). Game cards and maps were also made available at designated community distribution points (such as libraries, leisure centres, and other public venues) enabling anyone to join the game. A dedicated engagement coordinator worked in advance of the programme to build relationships with local anchor organisations, raising awareness and fostering participation through trusted local networks and memberships. The game also has a steering group made up of local partner organisations who help to organise the game, linked events, and connect the game to their networks and capacity.

On registering, participants were provided with information on data usage and asked to provide informed consent. Participants under 16 years were required to be registered by a parent, with consent from a parent/guardian necessary to engage in the intervention. All participants were welcome to participate in the initiative and there were no exclusion criteria for participation. The data reported here are a subset of this population geographically located in Chelmsford or South Woodham Ferrers based on their reported postcode. Ethical approval was granted for the analysis of this data from the Anglia Ruskin University ethical panel (Reference No: 2425–2808).

### Measures

Participants were asked to complete a baseline questionnaire after registering and before the six-week “game phase” of the intervention. Participants then completed a follow-up questionnaire applied at the end of the intervention. Survey responses were collected via an email link to a survey platform. Completion of the questionnaire was incentivised at baseline with 100 additional points and at follow up with participants entered into a prize draw for a gift voucher.

The questionnaires captured participant demographics, including age category, gender, ethnicity, and whether a participant had a long-term health condition (LTC, defined as one or more of diabetes, heart disease, COPD (emphysema), asthma or another long-term condition). or disability (defined as a condition that “which limits the activities or work you can do”). Deprivation was informed by index of multiple deprivation (IMD) decile determined from postcode provided at registration. Participants were deemed as from a deprived background if within IMD decile 1–4. The ethnicity question options were informed by the census 2021 list of ethnic groups [23], with participants identified as either white British or from an ethnically or culturally diverse background (ECD), with ECD defined as any responses that were not “white British”. the percentage of white British participants calculated.

Time spent sitting was estimated using the specific sedentary time questions from the short form international physical activity questionnaire (IPAQ-SF) [24]. The questions asked the amount of time in hours and minutes spent sitting or doing sedentary activities on an average weekday and weekend day. Total time spent sitting in hours on weekdays, weekend days and daily was calculated from these responses.

Time spent in MVPA, from which activity status was determined, was estimated subjectively using the relevant Active Lives Survey (Sport England) for children and adults. The questions are a seven-day recall of the time spent in hours and minutes in different activities and whether the time spent in the activities was of sufficient intensity to cause them to be ‘out of breath’ or ‘breathe faster’, therefore indicative of moderate or higher intensity activity [25]. The time spent in these activities (if of sufficient intensity) were summed to provide weekly minutes in MVPA. Activity status was identified according to Chief Medical Officer physical activity guidelines where adults were deemed inactive if engaged in < 150 min of MVPA/week or active if ≥ 150 min of MVPA/week. Children were classed as inactive if engagement in MVPA was on average < 420 min/week or ≥ 420 min/week for active, based on meeting the average 60 min of MVPA per day guideline on seven days of the week [26].

Mental wellbeing was indicated using the 14 item Warwick-Edinburgh Mental Wellbeing Scale (WEMWBS). The scale includes 14 positively worded accessible items covering aspects of feeling and functioning in the context of mental wellbeing. Responses are one of 5 options reflecting frequency a person experiences the feeling or functioning of the item, where responses range from ‘none of the time’ to ‘all of the time’. A single score was obtained from the scale by scoring item 1–5 based on their response then summing to produce a total score ranging from 14–70 [27].

Life satisfaction was measured in adults only using the Office of National Statistics’ question on life satisfaction, which asks ‘On a scale of 0–10, how satisfied are you with your life nowadays?’ [28]; where 0 is not at all satisfied with life and 10 is completely satisfied with life.

### Statistical analysis

Analyses were performed using IBM Statistical Package for Social Sciences (SPSS; IBM Corp. (2020). IBM SPSS Statistics for Windows (Version 27.0) [Computer software]. IBM Corp.). Raw data were screened for quality and filtered to those participants with complete data for the included outcomes. Descriptive statistics were produced for demographics and outcomes with demographics presented as percentage of the overall population of children or adults and measures presented as mean and standard deviation. Normality analyses were conducted with normality defined as skewness of ± 2 and kurtosis ± 7. Between-subjects baseline comparisons within demographic groups for all outcome measures were assessed using Kruskal–Wallis analyses followed by Dunn’s Test post-hoc analysis with Bonferroni adjustments. Mean differences were calculated to provide interpretations of differences. Within-subjects differences for all measures were assessed by Wilcoxon Signed Rank for children and adults, followed by each demographic grouping. Significance for all analyses was set at *p* < 0.05.

## Results

*N* = 13,893 participants engaged in the intervention, however, of these *n* = 11,494 provided necessary basic demographic information of age category, gender and ethnicity. Demographic breakdown of registered participants was 49% < 18 years, 61% female, and 69% adult female. *N* = 5244 unique participants (39% < 18 years, 66% female, 22% from an ethnically or culturally diverse background (ECD) completed questionnaire at baseline, with *n* = 1955 unique participants (39% < 18 years, 65% female, 18% ECD) at follow up. When matched *n* = 1344 provided both baseline and follow up questionnaires.

**Fig. 2:**
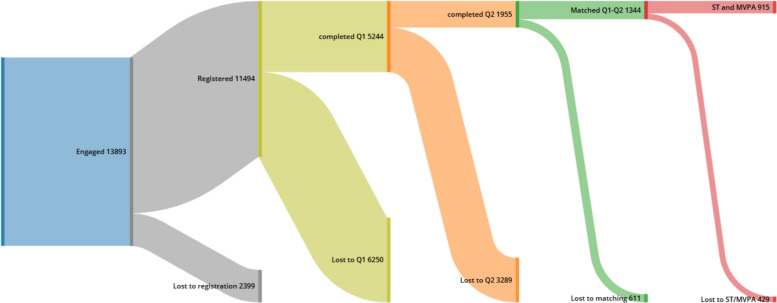
Participant flow-diagram showing proportion of participants in each stage of the study and where drop-out was recorded

*N* = 915 (*n* = 313 children) participants reported sitting time (ST) and MVPA both pre- and post-intervention, allowing detailed comparison. Figure 2 shows the breakdown of dropout to the reported population.

Descriptive statistics for this sub-population, used for subsequent analyses, are presented in Table 1, where data are presented as percentages of the population of children and adult groups.

**Table 1:** Demographic characteristics

%	Children (*n* = 313)	Adults (*n* = 602)
< 11 years	90.1	-
12–18 years	9.9	-
19–29 years	-	1.2
30–39 years	-	30.4
40–49 years	-	41.0
50–59 years	-	12.3
60–69 years	-	8.1
> 70 years	-	6.8
Female	59.1	73.9
Ethnically/Culturally Diverse (ECD)	17.3	13.8
Long-term health condition (LTC)	9.3	21.9
Reported Disability	3.5	4.8

*ECD* ethnically culturally diverse, *LTC* Long term health condition

Group comparison at baseline showed no differences in ST regardless of group for adults (*p* > 0.05). When groups were compared at baseline for children, under 11 s reported lower STdaily (X^2^ = 11.43 (1), *P* < 0.001), STweek (X^2^ = 12.41 (1), *P* < 0.001), STweekend (X^2^ = 7.42 (1), *P* < 0.01), and higher wellbeing scores (X^2^ = 5.89 (6), *P* < 0.05) compared to older children. For both children and adults overall, there was a pre- to post-intervention increase in reported wellbeing and a reduction in reported STdaily and STweek, while life satisfaction increased for adults overall. Additionally, the percentage of participants classed as inactive decreased for both children and adults (Table 2, *p* < 0.01).

**Table 2:** Sedentary time, wellbeing, and percentage inactive pre and post intervention

	Children (*n* = 314)		Adults (*n* = 695)	
	Pre	Post	Pre	Post
Weekday Sitting Time (STweek; hours)	5.84 ± 3.42	5.25 ± 3.10^*^	6.41 ± 3.59	5.70 ± 3.23^*^
Weekend Sitting Time (STweekend; hours)	5.39 ± 3.22	5.26 ± 3.41	5.32 ± 2.87	4.95 ± 2.79^*^
Daily Average Sitting Time (STdaily; hours)	5.62 ± 2.99	5.23 ± 3.04^*^	5.87 ± 2.85	5.33 ± 2.71^*^
Inactive (%)	63.7	57.6	38.1	28.5
Wellbeing Score	54.7 ± 9.3	57.1 ± 8.8^*^	48.1 ± 11.7	50.4 ± 11.0^*^
Life Satisfaction Score	-	-	7.15 ± 1.88	7.31 ± 1.97^*^

Mean ± SD, *MVPA* Moderate to vigorous physical activity

^*^Significant difference to pre at *p* < 0.05

When considering differences post intervention according to grouping for adults, STdaily was significantly reduced for females (−41 min; W = 6936, *p* < 0.01), those in their 30 s (−56 min; W = 12,471, *p* < 0.01) and 40 s (−29 min; W = 18,960, *p* < 0.01), with non-significant reductions for remaining groupings (*P* > 0.05).

STweek significantly reduced for males (−32 min; W = 6490, *p* < 0.01), females (−48 min; W = 54,686, *p* < 0.01), those in their 30 s (−62 min; W = 9828, *p* < 0.01) and 40 s (−43 min; W = 17,122, *p* < 0.01), with non-significant reductions for remaining groups (*p* > 0.05). Similarly, ST weekend reduced for females (−33 min; W = 51,895, *p* < 0.01), those in their 30 s (−49 min; W = 9974, *p* < 0.01) and 40 s (−16 min; W = 13,874, *p* < 0.05), with remaining groups reducing sitting time to a lesser extent (*p* > 0.05).

Wellbeing improved for males (+ 2.39; W = 6087, *p* < 0.01), females (+ 2.41; W = 538, *p* < 0.01), those in their 30 s (+ 2.52; W = 1362, *p* < 0.01) and 40 s (+ 2.55; W = 1360, *p* < 0.01). Life satisfaction also improved for females (+ 0.26; W = 15,166, *p* < 0.01) and for those in their 30 s (+ 0.28; W = 3524, *p* < 0.05) and 40 s (+ 0.09; W = 5087, *p* < 0.05). Wellbeing and life satisfaction trended towards an increase but was not statistically significant for remaining groups (*P* > 0.05).

Considering differences according to grouping for children, STdaily was reduced for girls (−23 min; W = 8872, *p* < 0.05), those aged 11 and under (−16 min; W = 19,518, *p* < 0.05), and adolescents (−74 min; W = 312, *p* < 0.05), with non-significant trends towards reductions in remaining groups (*p* > 0.05). STweek decreased significantly for girls (−33 min; W = 8229, *p* < 0.05) and approached significance for boys (−39 min; W = 3438, *p* = 0.057). Adolescents showed a larger decrease in STweek (−107 min; W = 306, *p* < 0.001), with younger children showing non-significant changes (*p* > 0.05). For STweekend, girls showed a modest reduction (−13 min; W = 7526, *p* < 0.05).

Wellbeing scores improved for both girls (+ 2.03; W = 4364, *p* = 0.001) and boys (+ 2.78; W = 1852, *p* = 0.004), as well as for under 11 s (+ 2.55; W = 9514, *p* < 0.001).

19.2% of adults and 16.6% of children moved from inactive to active. There were no differences in adult wellbeing with change in activity status, however those who moved from inactive to active trended towards improved wellbeing (+ 1.0; *p* = 0.46). Children who were active pre and remained active showed significant improvement in wellbeing (+ 2.6, *p* = 0.03), while children who moved from inactivity trended towards improvement (+ 1.2, *P* = 0.48; Table 3).

**Table 3:** Differences in wellbeing and life satisfaction for adults and children according to change in activity categories

Outcome	Activity Change	n	W	*P*-Value	Difference
Adult Wellbeing	In-In	127	29,098	0.628	−0.448
	Act-In	66	15,628	0.592	−0.675
	In-Act	131	28,158	0.461	1.026
	Act-Act	355	45,088	0.921	−0.158
Adult Life Satisfaction	In-In	127	28,429	0.455	0.099
	**Act-In**	**66**	**18,125**	**0.030**	**−0.479**
	In-Act	131	33,417	0.082	−0.234
	**Act-Act**	**355**	**43,315**	**0.042**	**0.243**
Child Wellbeing	In-In	148	11,889	0.103	−1.858
	Act-In	32	4618	0.136	−2.598
	In-Act	52	5716	0.483	1.213
	**Act-Act**	**81**	**7170**	**0.025**	**2.641**

CMO guidelines – inactive adults < 150 min/week, inactive children < 420 min/week

*In* Inactive, *Act* Active, *In-In* Inactive pre, inactive post, *Act-Act* Active pre, active post

For children, spearman’s correlation revealed no relationship between changes in STdaily, STweek, and STweekend with wellbeing. Regression analysis showed changes in STdaily (β = 0.023, *p* = 0.699), STweek (β = −0.055, *p* = 0.653) and STweekend (β = 8.4035 × 10^−9^, *p* = 1.000) did not predict wellbeing.

In adults, no correlation was found between any changes in ST and wellbeing, with similar found between any changes and life satisfaction. Regression analyses further confirmed the lack of significant predictive value of both STdaily change (β = − 0.035, *p* = 0.388), STweek change (β = − 0.062, *p* = 0.127) and STweekend change (β = 0.013, *p* = 0.752) on wellbeing change, with similar results found for life satisfaction.

## Discussion

The primary aims of this study were to evaluate the impact of a community intervention on ST and physical activity levels in one region of Essex. The main findings from this research are that the intervention was associated with reductions in ST and reductions in the proportion of inactive children and adults. Furthermore, the findings indicated improvements in wellbeing and life satisfaction. Taken together, these findings demonstrate that the Beat the Street intervention is a potentially effective intervention which may change behaviour in the short term in isolated locations such as Chelmsford and South Woodham Ferrers. To the authors knowledge this is this first study assessing the impact of a gamified community intervention including ST measures providing initial insight as to effective methods of changing community health behaviours. As such, the use of gamification in community settings could be a useful tool in targeting sedentary behaviours which are a key target amongst all populations given the decline of mental and physical health outcomes associated with the rise in sedentariness [29]. Of note, the WHO Guidelines on Physical Activity and Sedentary Behaviour were among the first to establish benchmark targets [30]. Children and adolescents are advised to accumulate no more than 2 h of sedentary behaviour per day, whereas adults are advised to break up prolonged sedentary behaviour by moving or at least standing every 30-min with additional physical activity when possible [30].

A key finding of the 6-week Beat the Street intervention was a positive impact on average STdaily for both children and adults, with a 23.4- and 32.4-min reduction per day, respectively. Such a reduction is likely to be clinically meaningful, especially in the adult population. For example, in one isotemporal substitution study including 851 adults residing in Sweden it was observed that replacing 30 min of ST each day with light-intensity physical activity was associated with significant reductions in all-cause mortality risk (HR: 0.89, 95% CI: 0.81–0.98) and CVD mortality risk (HR: 0.76, 95% CI: 0.63–0.92). Replacing 10 min of ST with moderate-to-vigorous physical activity was associated with reduction in CVD mortality risk (HR: 0.62, 95% CI: 0.42–0.91) [31]. Closer examination of the adult data demonstrates significant reductions in STdaily and STweekend for females, and those in their 30 s and 40 s. These are interesting findings and the reasons behind greater effects in these groups are elusive. Future research involving the Beat the Street intervention should incorporate qualitative components to gain a deeper understanding as to why the intervention elicited greater effects in specific groups. Interestingly, while the overall reduction in ST was notable, there were no significant reductions observed for older adults or males, suggesting the potential need for targeted interventions focusing on these specific groups. For example, targeted interventions may be required for older adults where accessibility issues associated to gamified interventions are a potential barrier.

For children, the results were more nuanced, with reductions in ST observed primarily among girls, children under the age of 11 years, and adolescents. Adolescents reported the largest decrease in STweek, likely because they typically begin with higher levels of sedentary behaviour and screen time, giving them greater scope for reduction over the 6-week intervention [32]. Younger children, on the other hand, showed minimal changes in their ST, suggesting that additional interventions might be necessary to address this age group more effectively, although it is noteworthy that sedentary behaviour in younger children is typically quite low in comparison to older children and adults.

Few studies have explored the impact of gamification on ST. However, there is a relatively large body of literature focusing on this topic in relation to physical activity. In a meta-analysis including 16 studies and 2407 participants a small to medium summary effect of gamified interventions on physical activity behaviour (g = 0.42 (95%CI [0.14, 0.69]) was observed. No statistical difference was found between different subgroups (adults vs adolescents, healthy participants vs adults with chronic diseases) and there were no interaction effects with moderators including age, gender or BMI, suggesting good scalability of gamified interventions. Moreover, the effect was stronger for step count (MD = + 1609.56 steps per day (95%CI [372.39, 2846.73]) than moderate-to-vigorous physical activity (g = 0.31 (95%CI [−0.19, 0.80]). The long-term effect (measured with follow-up averaging 14 weeks after the end of the intervention) was very small to small (g = 0.15, [0.07, 0.23]) [18]. Previous work by Mamede and colleagues [33] applied a 4-week gamification phase alongside social support but found little change in sedentary behaviour. This limited change was proposed to be a consequence of the bias associated with self-reported sedentary time, the short intervention period and the small sample size that responded [33]. In comparison, the present intervention observed significant changes in ST, highlighting that gamification has the potential to support short term changes in sedentary behaviour, a key target for public health interventions. However, the current research quantified total ST using self-report measures, which may have underestimated ST. Moreover, ST was quantified in total as opposed to identifying any patterns of breaks in this behaviour. Furthermore, interventions applying gamification to change sedentary behaviours applied similar principles of competition and incentivisation to trigger motivation to engage in moving more. Indeed, Mamede and colleagues [33] conducted their study in an office-based setting using only an app compared to the combination of physical and digital technology in a community-setting as a whole; whereas the present study changed the way participants engaged with their community and the built environment with the aim of increasing awareness of opportunity and promoting motivation. However, the long-term impact of Beat the Street on ST is unknown. Therefore, future research is necessary to understand the long-term impact as well the additional factors that influence engagement outside of gamification.

Our study also demonstrated decreases in the proportion of inactive children and adults. These findings align with previous research evaluating the effectiveness of Beat the Street on inactivity [34] and further research which found community-based gamification to effectively target short term physical activity changes [35]. Specifically, Harris and colleagues [34] found decreases of inactivity by 7% for adults across 18 locations, with changes proposed to be maintained for up to two years [36]. Comparatively, efforts to apply gamification in physical activity behaviour change has been less conclusive with mixed results and modest changes according to a recent review of RCTs [37]. Most research included in the review focused on the use of goal setting combined with progress or feedback to target increases in intrinsic motivation. In comparison the present intervention employed rewards, social collaboration and competition, thereby combining gamification with social incentives to motivate individuals and families to engage in physical activity. The positive outcomes observed in the present intervention compared to previous work suggests that the combination of gamification with wider social incentives may be more effective than gamification principles alone which may be considered in future work. The impact of Beat the Street in Chelmsford compared to national data further demonstrates the potential effectiveness with adult inactivity reduced to below the nationally reported level of 37% [7]. As such, these findings support that the application of gamification combined with wider incentives show promise to contribute to combatting inactivity at a larger population level, in support of literature showing the promise of promoting physical activity across populations [18] and aligns with the findings of Xu and colleagues [37]. Therefore, future research and interventions may consider combining gamification principles with wider social incentives in a community setting to encourage engagement with physical activity.

Wellbeing scores for adults and young people, as well as life satisfaction for adults, saw positive improvements during the intervention period and the positive effect of the intervention was more pronounced in adults than in children. However, the change in wellbeing and life satisfaction was not explained by the primary outcome of a decrease in ST. The limited predictive ability of ST is in line with previous research, and it was hypothesised that a greater magnitude of change in this behaviour or duration of intervention may be necessary to improve wellbeing. Specifically, Ellingson and colleagues [38] found reductions in ST across a year were predictive of improvements in wellbeing, with 60-min decreases necessary to prevent or reduce the negative effects of sedentary time on mental wellbeing. Alternatively, a recent review found little relationship between time in sedentary behaviours and wellbeing outcomes in adults, including life satisfaction [39]. Taken together, the evidence suggests the increase in wellbeing observed was likely a consequence of a combination of both physical activity and ST and wider changes associated with the Beat the Street intervention, including greater social engagement, connecting with nature and awareness of community assets.

## Limitations

There are some limitations which should be acknowledged which allow for our findings to be contextualised and provide opportunities for future work. First, the data included is from a single location with areas that are of relatively low deprivation which limits the generalisability of our findings, in particular places that experience high deprivation and a greater diversity of ethnicity and cultural backgrounds [40]. The limitation can be overcome in future work by conducting the intervention in multiple locations allowing for greater exploration of behaviour change in specific demographics. The authors are at present embarking on this work. Second, the subjective nature of the physical activity and sitting time measurement has been suggested to be less reliable than objective measurement due to social desirability biases. However, in this context the feasibility and practicality of using subjective measures outweighed the use of objective methods. Nevertheless, the limitations of these metrics should be acknowledged when interpreting the findings and future research could look to create a larger sample that will overcome the limitations of reliability and bias. Furthermore, the self-reported measures ST used in this study capture total time in this behaviour and do not provide specific detail as to the patterns by which such behaviours are accumulated. Next, this study has demonstrated the short-term impact of a gamification intervention on ST, however future efforts are needed to elucidate the longer-term impact in communities like Chelmsford, South Woodham Ferrers and further afield. Further limitations include the lack of a control group, as well as the use of incentives which could act as an external motivator for participants. Finally, there was a relatively high level of individuals engaging in the intervention but not data collection, and we did not explore the reasons behind this. We recommend that future Beat the Street interventions embed process evaluation into the study design to identify reasons behind non-engagement as well as adherence to inform the implementation in subsequent games.

## Conclusion

The present findings suggest that the Beat the Street intervention reduced ST and increased physical activity among both children and adults in the short term; by combining gamification, such as points, rewards, and friendly competition, with social incentives like group challenges and community engagement. Further research is needed to assess the long-term impacts and explore objective measures of sedentary and physical activity behaviours to better understand sustained engagement and outcomes.

## Data Availability

The data that support the findings of this study are available from Intelligent Health but restrictions apply to the availability of these data, which were used under license for the current study, and so are not publicly available. Data are however available from the authors upon reasonable request and with permission of Intelligent Health. Requests should be directed to Steve Rose at [steve.rose@intelligenthealth.co.uk] (mailto:steve.rose@intelligenthealth.co.uk).

## Abbreviations

ECD: Ethnically or culturally diverse background
IPAQ-SF: International physical activity questionnaire (short form)
IMD: Index of multiple deprivation
LTC: Long term health condition
MVPA: Moderate to vigorous physical activity
ST: Sitting time
STweek: Weekday sitting time
STweekend: Weekend sitting time
STdaily: Daily average sitting time

## References

[1] Posadzki P, Pieper D, Bajpai R, Makaruk H, Könsgen N, Neuhaus AL, et al. Exercise/physical activity and health outcomes: an overview of cochrane systematic reviews. BMC Public Health. 2020;20(1):1724. 10.1186/s12889-020-09855-3 PMC7670795 33198717

[2] Rahmati M, Lee S, Yon DK, Lee SW, Udeh R, McEvoy M, et al. Physical activity and prevention of mental health complications: an umbrella review. Neurosci Biobehav Rev. 2024;160:105641. Available from: https://www.sciencedirect.com/science/article/pii/S0149763424001106. 38527637 10.1016/j.neubiorev.2024.105641

[3] Caspersen CJ, Powell KE, Christenson GM. Physical activity, exercise, and physical fitness: definitions and distinctions for health-related research. Public Health Rep. 1985;100(2):126–31. 3920711 PMC1424733

[4] Tremblay MS, Aubert S, Barnes JD, Saunders TJ, Carson V, Latimer-Cheung AE, et al. Sedentary Behavior Research Network (SBRN) – terminology consensus project process and outcome. Int J Behav Nutr Phys Act. 2017;14(1):75. 10.1186/s12966-017-0525-8 PMC5466781 28599680

[5] Park JH, Moon JH, Kim HJ, Kong MH, Oh YH. Sedentary lifestyle: overview of updated evidence of potential health risks. Korean J Fam Med. 2020;41(6):365–73. 33242381 10.4082/kjfm.20.0165 PMC7700832

[6] GOV.UK. Physical activity guidelines: children and young people (5 to 18 years). GOV.UK; 2019. Available from: https://www.gov.uk/government/publications/physical-activity-guidelines-children-and-young-people-5-to-18-years.

[7] Sport England. Adults’ activity levels in England bounce back to pre-pandemic levels. Sport England; 2023. Available from: https://www.sportengland.org/news/adults-activity-levels-england-bounce-back-pre-pandemic-levels.

[8] Office for Health Improvement & Disparities. Physical activity data tool: statistical commentary, January 2022. GOV.UK; 2022. Available from: https://www.gov.uk/government/statistics/physical-activity-data-tool-january-2022-update/physical-activity-data-tool-statistical-commentary-january-2022.

[9] Hamer M, Stamatakis E, Chastin S, Pearson N, Brown M, Gilbert E, et al. Feasibility of measuring sedentary time with thigh worn accelerometry, and sociodemographic correlates: the 1970 British cohort study. Am J Epidemiol. 2020;189(9):963-71. 10.1093/aje/kwaa047 PMC7443760 32219368

[10] Hesketh KR, Brage S, Inskip HM, Crozier SR, Godfrey KM, Harvey NC, et al. Activity behaviors in British 6-year-olds: cross-sectional associations and longitudinal change during the school transition. J Phys Act Health. 2022;19(8):558–65. 35894892 10.1123/jpah.2021-0718 PMC7613624

[11] Sport England. Active Lives and Active People Surveys 2023-2024. 6th Release. UK Data Service, 2025.

[12] Sahlqvist S, Song Y, Ogilvie D. Is active travel associated with greater physical activity? The contribution of commuting and non-commuting active travel to total physical activity in adults. Prev Med. 2012;55(3):206–11. 22796629 10.1016/j.ypmed.2012.06.028 PMC3824070

[13] Roth MA, Millett CJ, Mindell JS. The contribution of active travel (walking and cycling) in children to overall physical activity levels: a national cross sectional study. Prev Med. 2012;54(2):134–9. 22182478 10.1016/j.ypmed.2011.12.004

[14] van Sluijs EMF, Fearne VA, Mattocks C, Riddoch C, Griffin SJ, Ness A. The contribution of active travel to children’s physical activity levels: cross-sectional results from the ALSPAC study. Prev Med. 2009;48(6):519–24. 19272404 10.1016/j.ypmed.2009.03.002 PMC3839265

[15] Smith L, Sahlqvist S, Ogilvie D, Jones A, Griffin SJ, van Sluijs E. Is active travel to non-school destinations associated with physical activity in primary school children? Prev Med. 2012;54(3–4):224–8. 22285945 10.1016/j.ypmed.2012.01.006 PMC3856476

[16] Skarin F, Olsson LE, Friman M, Wästlund E. Importance of motives, self-efficacy, social support and satisfaction with travel for behavior change during travel intervention programs. Transp Res Part F Traffic Psychol Behav. 2019;62:451–8.

[17] Nishi SK, Kavanagh ME, Ramboanga K, Ayoub-Charette S, Sébastien Modol, Dias GM, et al. Effect of digital health applications with or without gamification on physical activity and cardiometabolic risk factors: a systematic review and meta-analysis of randomized controlled trials. EClinicalMedicine. 2024;76:102798–8. 10.1016/j.eclinm.2024.102798 PMC11701442 39764571

[18] Mazeas A, Duclos M, Pereira B, Chalabaev A. Does gamification improve physical activity? A systematic review and meta-analysis of randomised controlled trials. J Med Internet Res. 2022;24(1):e26779. 10.2196/26779 PMC8767479 34982715

[19] Buttazzoni A, Nelson Ferguson K, Gilliland J. Barriers to and facilitators of active travel from the youth perspective: a qualitative meta-synthesis. SSM Popul Health. 2023;22:101369. 36909930 10.1016/j.ssmph.2023.101369 PMC9996358

[20] Cole R, Leslie E, Donald M, Cerin E, Neller A, Owen N. Motivational readiness for active commuting by university students: incentives and barriers. Health Promot J Austr. 2008;19(3):210–5. 19053938 10.1071/he08210

[21] BTS - Homepage. beatthestreet.me. Available from: https://beatthestreet.me/.

[22] Coombes E, Jones A. Gamification of active travel to school: a pilot evaluation of the Beat the Street physical activity intervention. Health Place. 2016;39:62–9. 26974232 10.1016/j.healthplace.2016.03.001 PMC5405045

[23] GOV.UK. List of ethnic groups. GOV.UK; 2021. Available from: https://www.ethnicity-facts-figures.service.gov.uk/style-guide/ethnic-groups/.

[24] Lee PH, Macfarlane DJ, Lam T, Stewart SM. Validity of the international physical activity questionnaire short form (IPAQ-SF): a systematic review. Int J Behav Nutr Phys Act. 2011;8(1):115. 22018588 10.1186/1479-5868-8-115 PMC3214824

[25] Sport England. Active Lives and Active People Surveys. [data series]. 5th Release. UK Data Service. SN: 2000120. 2023. 10.5255/UKDA-Series-2000120.

[26] Department of Health and Social Care. Physical activity guidelines: UK Chief Medical Officers’ report. Gov.uk. Department of Health and Social Care; 2019. Available from: https://www.gov.uk/government/publications/physical-activity-guidelines-uk-chief-medical-officers-report.

[27] Tennant R, Hiller L, Fishwick R, Platt S, Joseph S, Weich S, et al. The Warwick-Edinburgh Mental Well-Being Scale (WEMWBS): development and UK validation. Health Qual Life Outcomes. 2007;5(1):63. Available from: https://hqlo.biomedcentral.com/articles/10.1186/1477-7525-5-63. 18042300 10.1186/1477-7525-5-63 PMC2222612

[28] Rees E, Eddolls J. Surveys using our four personal well-being questions - Office for National Statistics. Ons.gov.uk; 2018. Available from: https://www.ons.gov.uk/peoplepopulationandcommunity/wellbeing/methodologies/surveysusingthe4officefornationalstatisticspersonalwellbeingquestions.

[29] Goyal J, Rakhra G. Sedentarism and chronic health problems. Korean J Fam Med. 2024;45(5):239–57. 39327094 10.4082/kjfm.24.0099 PMC11427223

[30] World Health Organization. WHO guidelines on physical activity and sedentary behaviour. 2020. Available from: https://iris.who.int/bitstream/handle/10665/336656/9789240015128-eng.pdf?sequence=1. 33369898

[31] Dohrn IM, Kwak L, Oja P, Sjöström M, Hagströmer M. Replacing sedentary time with physical activity: a 15-year follow-up of mortality in a national cohort. Clin Epidemiol. 2018. Available from: https://www.dovepress.com/replacing-sedentary-time-with-physical-activity-a-15-year-follow-up-of-peer-reviewed-fulltext-article-CLEP. 10.2147/CLEP.S151613 PMC5790069 29416378

[32] Boone JE, Gordon-Larsen P, Adair LS, Popkin BM. Screen time and physical activity during adolescence: longitudinal effects on obesity in young adulthood. Int J Behav Nutr PhysAct. 2007;4(1):26. Available from: https://ijbnpa.biomedcentral.com/articles/10.1186/1479-5868-4-26. 10.1186/1479-5868-4-26 PMC1906831 17559668

[33] Mamede A, Noordzij G, Jongerling J, Snijders M, Schop-Etman A, Denktas S. Combining web-based gamification and physical nudges with an app (MoveMore) to promote walking breaks and reduce sedentary behavior of office workers: field study. J Med Internet Res. 2021;23(4):e19875. 33843593 10.2196/19875 PMC8076996

[34] Harris MA, Bird W. Bright spots, physical activity investments that work: beat the Street. Br J Sports Med. 2020;54(8):489-90. 10.1136/bjsports-2018-099992 30323058

[35] Liu W, Ligmann-Zielinska A. A pilot study of Pokémon Go and players’ physical activity. Games Health J. 2017;6(6):343–50. 28853912 10.1089/g4h.2017.0036

[36] Harris MA. Maintenance of behaviour change following a community-wide gamification based physical activity intervention. Prev Med Rep. 2019;13:37–40. 30510892 10.1016/j.pmedr.2018.11.009 PMC6260258

[37] Xu L, Shi H, Shen M, Ni Y, Zhang X, Pang Y, et al. The effects of mHealth-based gamification interventions on participation in physical activity: systematic review. JMIR Mhealth Uhealth. 2022;10(2):e27794. 35113034 10.2196/27794 PMC8855282

[38] Ellingson LD, Meyer JD, Shook RP, Dixon PM, Hand GA, Wirth MD, et al. Changes in sedentary time are associated with changes in mental wellbeing over 1 year in young adults. Prev Med Rep. 2018;11(1):274–81. Available from: https://www.sciencedirect.com/science/article/pii/S2211335518301268. 30116698 10.1016/j.pmedr.2018.07.013 PMC6082791

[39] Sui W, Sui A, Prapavessis H. Relationships between indices of sedentary behavior and hedonic well-being: a scoping review. Psychol Sport Exerc. 2021;54:101920.

[40] Indices of Multiple Deprivation (IMD) 2019 full report. Essex County Council. Available from: https://data.essex.gov.uk/dataset/indices-of-multiple-deprivation-imd-2019-full-report-2w89n.

